# Resveratrol Alleviates Zearalenone-Induced Intestinal Dysfunction in Mice through the NF-κB/Nrf2/HO-1 Signalling Pathway

**DOI:** 10.3390/foods13081217

**Published:** 2024-04-17

**Authors:** Sugan Xia, Chaoyue Yan, Jianhong Gu, Yan Yuan, Hui Zou, Zongping Liu, Jianchun Bian

**Affiliations:** 1 College of Veterinary Medicine, Yangzhou University, Yangzhou 225009, China; xiasugan07@163.com (S.X.); yanchaoyue0407@163.com (C.Y.); jhgu@yzu.edu.cn (J.G.); yuanyan@yzu.edu.cn (Y.Y.); zouhui@yzu.edu.cn (H.Z.); liuzongping@yzu.edu.cn (Z.L.); 2 Jiangsu Co-Innovation Center for Prevention and Control of Important Animal Infectious Diseases and Zoonoses, Yangzhou 225009, China; 3 Joint International Research Laboratory of Agriculture and Agri-Product Safety, Ministry of Education of China, Yangzhou University, Yangzhou 225009, China

**Keywords:** resveratrol, zearalenone, inflammation, oxidative stress, intestinal barrier

## Abstract

Zearalenone (ZEA), a mycotoxin widely present in crops and food, poses a major threat to animal and human health. The consumption of ZEA-contaminated food or feed causes intestinal damage. Therefore, exploring how to mitigate the intestinal damage caused by its ZEA is becoming increasingly important. Resveratrol (RSV), a polyphenol compound, mainly exists in *Vitis vinifera*, *Polygonum cuspidatum*, *Arachis hypogaea*, and other plants. It has potent anti-inflammatory and antioxidant activity. The primary objective of this study was to assess the defensive effects of RSV and its molecular mechanism on the intestinal mucosal injury induced by ZEA exposure in mice. The results showed that RSV pretreatment significantly reduced serum DAO and that D-lactate levels altered intestinal morphology and markedly restored TJ protein levels, intestinal goblet cell number, and MUC-2 gene expression after ZEA challenge. In addition, RSV significantly reversed serum pro-inflammatory factor levels and abnormal changes in intestinal MDA, CAT, and T-SOD. Additional research demonstrated that RSV decreased inflammation by blocking the translocation of nuclear factor-kappaB (NF-κB) p65 and decreased oxidative stress by activating the nuclear factor E2-related factor 2 (Nrf2) pathway and its associated antioxidant genes, including NQO1, γ-GCS, and GSH-PX. In summary, RSV supplementation attenuates intestinal oxidative stress, inflammation, and intestinal barrier dysfunction induced by ZEA exposure by mediating the NF-κB and Nrf2/HO-1 pathways.

## 1. Introduction

ZEA, also known as F-2 toxin, is a mycotoxin produced mainly by scab and Fusarium in maize, with oestrogen-like effects [1,2]. Mycotoxin contamination of crops and food is a global problem, particularly in maize and wheat. This leads to significant financial damages in tainted crops while presenting a possible danger to the health of livestock and humans [3]. Based on global survey data from the past 10 years, the detection rate of ZEA in grains is approximately 46%, with the highest recorded content being 3049 µg/kg [4]. According to European regulations, the maximum level of ZEA in edible oil is 400 μg/kg [5]. Previous investigations have shown that exposure to ZEA causes severe health problems in animals, including reproductive and immune toxicity [6,7]. In addition, ZEA also damages the intestinal barrier. Initial research has indicated that ZEA administered at levels of 1.0 and 5.0 mg/kg can impact the structure of the intestines and weaken the integrity of the intestinal barrier by decreasing the presence of tight junction (TJ) proteins [8].

The intestinal barrier consists of a chemical, physical, and immunological barrier. The primary defense, consisting of the mucosal layer, intestinal epithelial cells, and tight junctions at the membrane junction, is crucial. TJs mainly include claudin, occludin, and ZO families. Previous studies have suggested that the NF-κB and Nrf2 signalling pathways play an essential role in the lysis of TJs and the regulation of paracellular permeability [9,10,11,12], their participation in ZEA-induced intestinal barrier destruction remains unclear. The NF-κB and Nrf2 signalling pathways are potential targets of RSV for treating ZEA-induced damage to the intestinal barrier. The mucous membrane layer comprises mucins, digestive enzymes, anti-microbial peptides, and immunoglobulins [13]. Goblet cells in the bowel are responsible for the production of mucus. Research has demonstrated a direct correlation between abnormal mucin expression and various gastrointestinal conditions [14,15].

RSV is a phytoalexin present in various plants, especially grapes, peanuts, and hellebores [16,17]. Several studies have shown that RSV possesses multiple pharmacological activities, including anti-inflammatory, antioxidant, and immunoregulatory effects [18,19,20,21]. RSV decreased intestinal barrier damage in a mouse model of colitis caused by DSS and in intestinal Caco-2 cells by blocking NF-κB signalling [20]. Prior research has indicated that oxidative harm plays a crucial role in the toxicity of intestinal cells induced by ZEA. Pretreating IPEC-J2 cells with RSV protected them from oxidative stress through the Nrf2 pathway, reduced intracellular reactive oxygen species (ROS) and apoptosis rates, and upregulated the intestinal barrier [21]. Dietary resveratrol supplementation can improve growth performance and reduce liver inflammation by increasing Nrf2 and HO-1 mRNA levels and reducing TLR4 and NF-κB mRNA levels in the liver of ducks [22].

Building on prior research, this study seeks to determine if RSV can protect against intestinal oxidative stress, inflammation, and barrier damage caused by ZEA in mice. In addition, the mechanism of RSV on ZEA-induced intestinal injury was explored through the protection of mucosa and the regulation of NF-κB and Nrf2 signalling pathways.

## 2. Materials and Methods

### 2.1. Animals

Male BALB/c mice (*n* = 40, five weeks of age) were purchased from the College of Comparative Medicine of Yangzhou University (Yangzhou, Jiangsu Province, China). It has been shown that ZEA absorption and intestinal glucuronidation are higher in males than in females [23]. Therefore, we used only male mice to avoid potentially influencing sex differences and fluctuations of sex hormones in female mice. Temperature (22–25 °C) and humidity (50–70%) were controlled in an adequately ventilated room, and all mice were fed sterile standard chow and sterile drinking water during their stay. Following a week of adjustment, the mice were allocated randomly to five categories: (1) control (*n* = 8); (2) 40 mg/kg body weight (BW) ZEA (*n* = 8); (3) 40 mg/kg BW ZEA+ 50 mg/kg BW RSV (*n* = 8); (4) 40 mg/kg BW ZEA+ 100mg/kg BW RSV (*n* = 8); and (5) 40 mg/kg BW ZEA+ 200 mg/kg BW RSV (*n* = 8). The experiment lasted for 12 days. Mice in different groups were administered either phosphate buffer solution (PBS) or RSV supplements orally daily for 12 days. Additionally, mice in each group were orally treated with PBS or ZEA for 4–8 days. On the 12th day, the mice were euthanised to collect jejunum tissue and serum samples, which were then stored at −80 °C until further analysis. All animal experiments were approved by the Yangzhou University Animal Care and Use Committee under Approval ID SYXK (Su) 2017-0044.

### 2.2. Transmission Electron Microscopy (TEM)

TEM examined the ultrastructure of jejunal tissue. Jejunal tissue was chopped into small pieces, then treated with 2.5% glutaraldehyde and 1% osmic acid for 1–2 h, dehydrated using a gradient of ethanol (50–100%), and finally encased in resin. Finally, 60 nm ultrathin sections were stained with uranyl acetate and lead citrate and examined under TEM (H-7650, Hitachi, Tokyo, Japan).

### 2.3. Periodic Acid-Schiff (PAS) Staining 

PAS was used to stain the tissue sections. The sections were then deparaffinised in xylene and rehydrated through an ethanol gradient. Following exposure to 1% periodic acid (Service Biotech, Wuhan, China) for 15 min, the sections were washed once with running tap water, then twice with distilled water, and placed in Schiff reagent (Service Biotech) for 30 min under controlled lighting. The sections were treated with hematoxylin, distinguished, dried, and put on a mount. Images were captured using a light microscope (BX53, Olympus, Tokyo, Japan) equipped with a computer-supported imaging system.

### 2.4. Hematoxylin and Eosin (H&E) Staining and Immunostaining

Jejunum tissue was harvested and immediately fixed in 4% neutral buffered paraformaldehyde solution. To evaluate the tissue samples histopathologically and measure the length of villi and depth of crypts in the intestines, they were embedded in paraffin, sectioned into 6 μm slices, and stained with H&E dye. Jejunum samples were analyzed under a light microscope, and the length of villi and depth of crypts were quantified with Image-Pro Plus 6.0 software.

The jejunum Nrf2, NF-κB, and TJ (occludin, claudin-1, and ZO-1) were examined using immunohistochemical analysis to determine their expression and distribution. The sections were treated with a 1% bovine serum albumin (BSA) solution for an hour before being exposed to antibodies overnight at a temperature of 4 °C. Sections were then incubated with secondary antibodies, followed by the addition of diaminobenzidine (DAB). The sections were then stained with haematoxylin. They were dehydrated through an ethanol gradient (70–100%) and treated with xylene to increase transparency. Lastly, the sections were observed under an optical microscope.

For immunofluorescence staining, jejunum sections were deparaffinised in xylene and graded alcohol. After a PBS wash, sections were incubated with 5% BSA for 1 h at room temperature. Next, the sections were exposed to the primary antibody NF-κB p65 and incubated overnight at 4 °C. Afterwards, the sections were treated with fluorescent secondary antibodies for an hour at 37 °C and stained with 4′,6-diamidino-2-phenylindole (DAPI). A microscope (Olympus, Tokyo, Japan) was used to observe and photograph the jejunum samples. In each sample, six optical areas were taken randomly for analysis.

### 2.5. Biochemical Assay

ELISA kits from Shanghai Hengyuan Biological in Shanghai, China, were used to measure serum levels of inflammatory cytokines’ tumour necrosis factor-α (TNF-α), interleukin-1β (IL-1β), and interleukin-6 (IL-6) following the manufacturer’s instructions. Furthermore, kits from Nanjing Jiancheng in China were utilised to analyze the MDA, CAT, and T-SOD levels in the jejunal homogenate. Serum DAO and D-lactate levels were assessed with ELISA kits (Meiliang, Shanghai, China), following the manufacturer’s guidelines.

### 2.6. Quantitative Rt-PCR

Jejunal tissue extracted total RNA using Trizol reagent (Invitrogen, Waltham, MA, USA) following the manufacturer’s guidelines. RNA was reverse transcribed to cDNA using the reverse transcription kit script QRT supermax for qPCR (Vazyme, Nanjing, China). The T043 NanoDrop (Thermo, MA, USA) was utilised to determine the concentration. The qRTPCR analysis was conducted on an Applied Biosystems 7500 RT-PCR machine (Foster City, CA, USA) with ChamQ SYBR qPCR Master Mix (Vazyme). The quantitative analysis followed Pfaffl’s mathematical model and utilised the 2^−ΔΔCt^ formula. The primers were produced by Sangon (Shanghai, China), and the sequences are shown in Table 1.

### 2.7. Western Blot Analysis

RIPA buffer (Beyotime Biotechnology, Shanghai, China) containing 1 mM PMSF (New Cell & Molecular Biotech, Suzhou, China) was used to homogenise and lyse the jejunal tissue. A BCA protein assay kit (Vazyme Biotech Co., Ltd.) was used to measure the total protein concentration. Protein extracts were combined with 5× SDS-PAGE loading buffer from Vazyme Biotech Co., Ltd. and heated in boiling water to denature. Subsequently, protein extracts from every sample were applied to 10% SDS-PAGE gels and then transferred to PVDF membranes. Primary antibodies were used to membranes and incubated overnight at 4 °C following a 2-h block with 5% nonfat dry milk at 37 °C. Primary antibodies included anti-NF-κB (p65) (Rabbit, 1:1000, Cell Signalling Technology, Danvers, MA, USA), anti-p-NF-κB (p-p65) (Rabbit,1:1000, Cell Signalling Technology, Danvers, MA, USA), anti-HO-1 (Rabbit, 1:1000, Cell Signalling Technology, Danvers, MA, USA), anti-Nrf2 (Rabbit, 1:1000, Cell Signalling Technology, Danvers, MA, USA), anti-ZO-1 (Rabbit, 1:2000, Proteintech, Wuhan, China), anti-occludin (Rabbit, 1:1000, Abcam, Cambridge, MA, USA), anti-claudin-1 (Rabbit, 1:2000, Proteintech, Wuhan, China) and anti-β-actin (Mouse, 1:20,000, Proteintech, Wuhan, China). Subsequently, secondary antibodies (1:10,000, Jackson ImmunoResearch, Lancaster, PA, USA) were applied to the membranes, followed by visualisation of the antigen-antibody reaction using enhanced chemiluminescence (ECL, New Cell & Molecular Biotech, China).

### 2.8. Data Analysis

Results are shown as average ± standard deviation, with each experiment conducted in three replicates. Data analysis was performed using one-way ANOVA multiple comparisons, comparing the mean of each column with the mean of every other column. Tukey’s statistical hypothesis testing was employed to adjust for multiple comparisons. Statistically significant differences were determined with a *p*-value less than 0.05. GraphPad Prism 8 software was utilised for data analysis and creating statistical graphics.

## 3. Results

### 3.1. RSV Prevents Intestinal Morphological Changes Caused by ZEA in Mice

**Figure 1:**
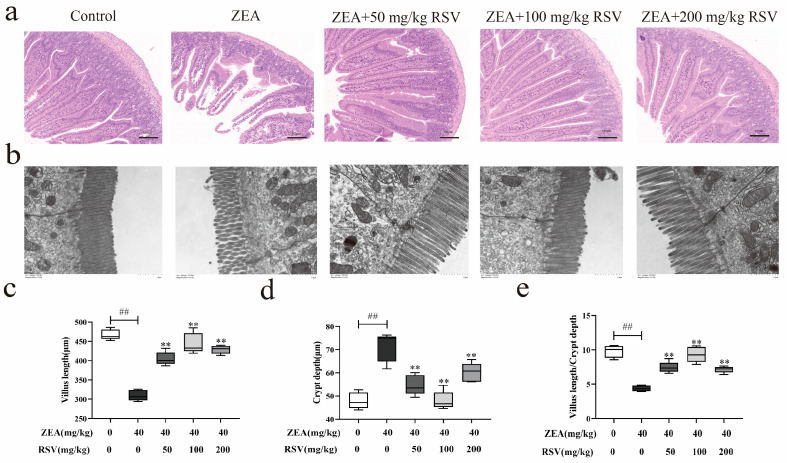
Effect of RSV on restoring intestinal epithelial injury in ZEA-treated mice. (**a**) H&E staining, ×100 of jejunum tissue. (**b**) TEM, ×10,000 of jejunum tissue. (**c**–**e**) Measurements were taken to determine the villi’s height, the crypts’ depth, and the ratio of villus length to crypt depth in the jejunum (*n* = 6). Values are presented as the mean ± SD of each treatment. “^##^” *p* < 0.01 compared to control group; “**” *p* < 0.01 compared ZEA group.

To study how RSV impacts the morphological changes in the intestines of mice induced by ZEA, the alterations in the pathological sections were evaluated using TEM and H&E staining. In Figure 1a, it is evident that ZEA treatment led to a notable decrease in the length of intestinal villi and caused changes in the shape and composition of the intestine compared to the control group. After RSV pretreatment, the intestinal villi height increased, and the morphology and structure of the intestines significantly improved. TEM analysis showed that ZEA destroyed the TJ structure in the jejunum, which was firmly fixed by RSV treatments (Figure 1b). Furthermore, the analysis of villus length and crypt depth indicated a significant increase in villus length and a decrease in crypt depth with RSV compared to the ZEA treatment group. Furthermore, the villus length and crypt depth in the jejunum showed a significant increase in the groups treated with RSV compared to the ZEA group. The findings indicate that RSV helps reduce the damage to intestinal tissue caused by ZEA (Figure 1c–e).

### 3.2. RSV Alleviates the Upregulation of Intestinal Permeability Induced by ZEA 

**Figure 2:**
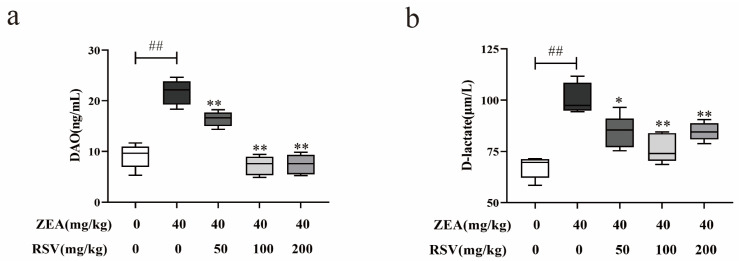
Effect of RSV on intestinal permeability in mice treated with ZEA. (**a**) The serum DAO, and (**b**) The serum D-lactate (*n* = 6). Values are presented as the mean ± SD of each treatment. “^##^” *p* < 0.01 compared to control group; “*” *p* < 0.05 and “**” *p* < 0.01 compared ZEA group.

To study how RSV affects the alterations in intestinal permeability caused by ZEA in mice, the concentrations of D-lactate and DAO were measured using ELISA. The findings indicated that RSV led to a notable reduction in the concentrations of D-lactate and DAO, which had been increased by ZEA exposure (Figure 2a,b).

### 3.3. RSV Alleviates Intestinal Chemical Barrier Impairment in ZEA-Treated Mice

**Figure 3:**
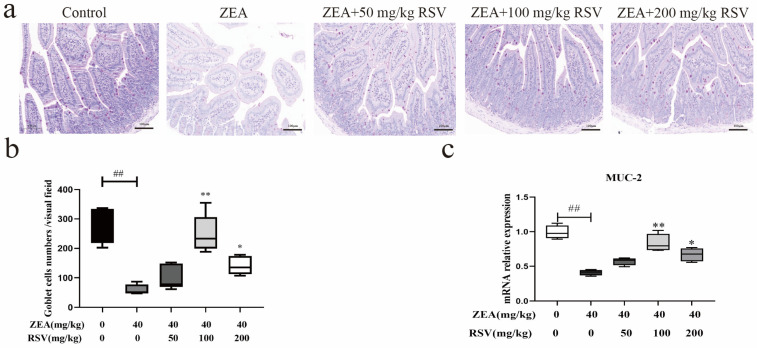
Effect of RSV on intestinal chemical barrier in ZEA-exposed mice. (**a**) Jejunum goblet cells are shown by PAS staining. (**b**) the number of jejunum goblet cells (*n* = 6). (**c**) mRNA expression of MUC-2 (*n* = 6). Values are presented as the mean ± SD of each treatment. “^##^” *p* < 0.01 compared to control group; “*” *p* < 0.05 and “**” *p* < 0.01 compared ZEA group.

To investigate how RSV impacts the chemical barrier of the intestine affected by ZEA in mice, we assessed the number of goblet cells and the proportion of MUC-2 mRNA expression in the epithelium of the jejunum. The results showed that RSV supplementation reversed the ZEA-induced decrease in the jejunal goblet cell number. (Figure 3a,b). Similarly, RSV reversed the ZEA-induced reduction in MUC-2 mRNA expression in the jejunum (Figure 3c).

### 3.4. RSV Increases the Expression of the TJ Protein in the Intestine of Mice Treated with ZEA

**Figure 4:**
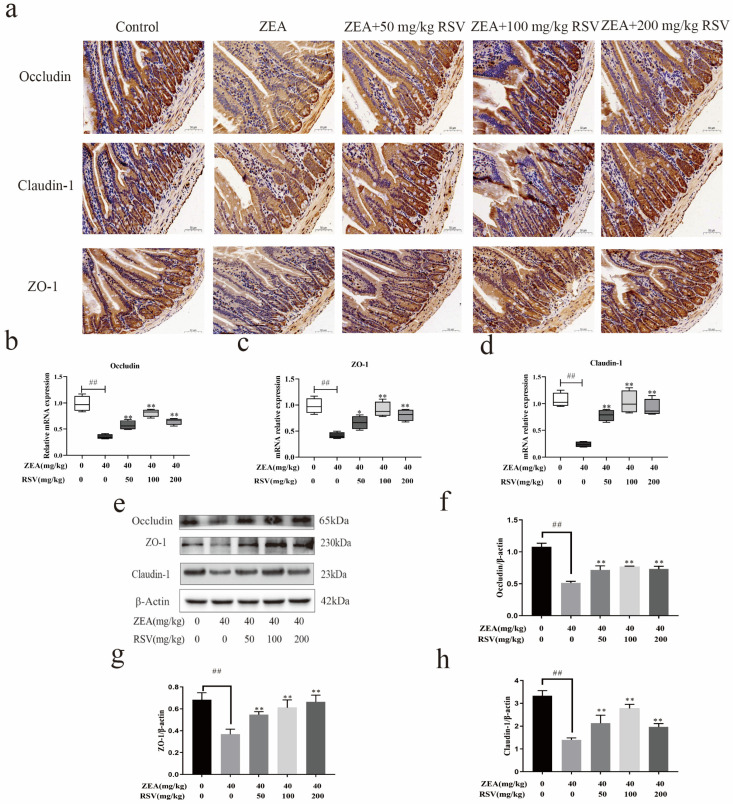
Impact of RSV on the gut barrier impairment caused by ZEA in mice. (**a**) Analysis using immunohistochemistry was performed on occludin, ZO-1, and claudin-1 in the epithelial cells of the jejunum in mice (magnification: 100× and scale bar: 50 μm). (**b**–**d**) mRNA levels of occludin, claudin-1, and ZO-1 were measured (*n* = 6). (**e**–**h**) Western blot analysis of occludin, claudin-1, and ZO-1 protein levels and relative intensity calculations (*n* = 3). Values are presented as the mean ± SD of each treatment. “^##^” *p* < 0.01 compared to control group; “*” *p* < 0.05 and “**” *p* < 0.01 compared ZEA group.

To investigate how RSV impacts the physical barrier created by ZEA in the jejunum of mice, the levels and localisation of occludin, claudin-1, and ZO-1 proteins were analyzed. An analysis using qRT-PCR and Western blotting showed that exposure to ZEA decreased the levels of occludin, claudin-1, and ZO-1 in the intestines compared to the control group. Elevated levels of occludin, claudin-1, ZO-1 mRNA,3, and protein were observed following RSV pre-treatment (Figure 4b–h). Immunohistochemical analysis indicated that ZEA caused a significant decrease in the protein expression of ZO-1, occludin, and claudin-1, while RSV effectively mitigated these impacts.

### 3.5. RSV Regulates ZEA-Triggered Inflammatory Cytokine Secretion in Mice

**Figure 5:**
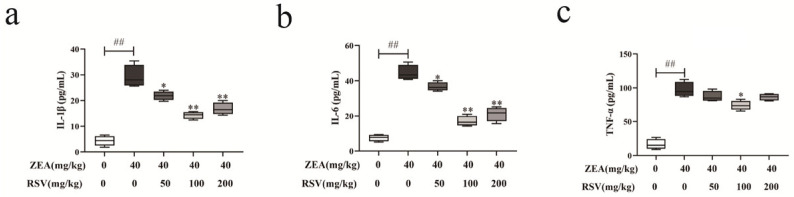
Effect of RSV on serum cytokines in ZEA-exposed mice (*n* = 6). ELISA was used to evaluate the concentrations of (**a**) TNF-α, (**b**) IL-1β, and (**c**) IL-6 in the serum of mice. Values are presented as the mean ± SD of each treatment. “^##^” *p* < 0.01 compared to control group; “*” *p* < 0.05 and “**” *p* < 0.01 compared ZEA group.

An ELISA was performed to verify the effect of RSV on ZEA-induced inflammatory cytokines in mice serum. Following exposure to ZEA, the serum concentrations of TNF-α, IL-1β, and IL-6 were elevated compared to the control group mice (see Figure 5a–c). RSV-pretreated mice showed a significant reduction of these cytokines at the same time.

### 3.6. RSV Relieves ZEA-Induced Intestinal Damage through the NF-κB Signalling Pathway

**Figure 6:**
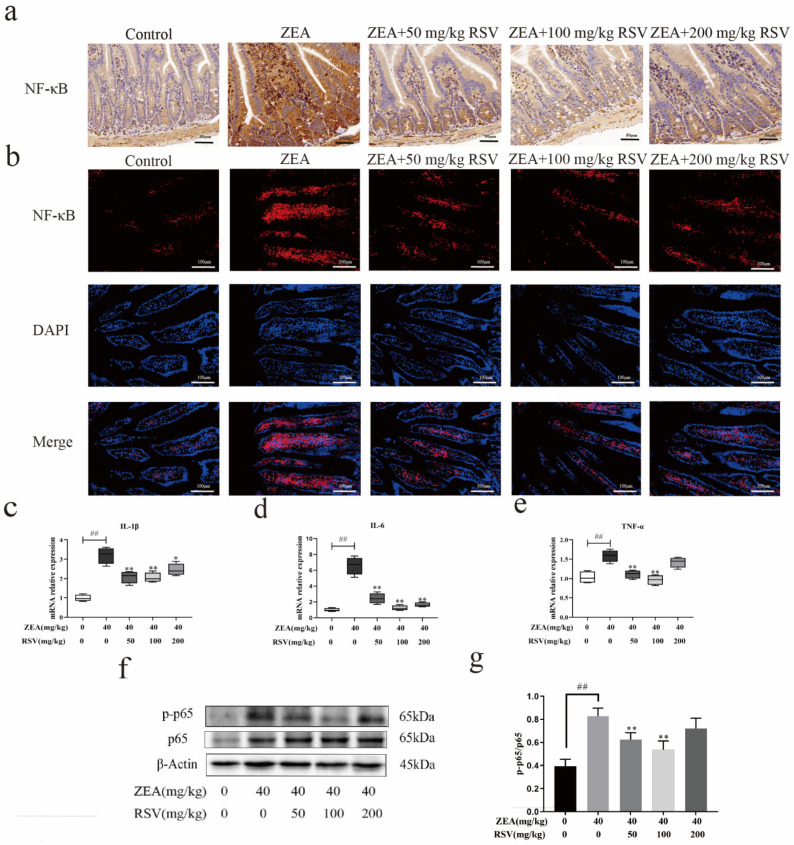
Impact of RSV on the NF-κB signalling pathway during ZEA-induced intestinal inflammation in mice. (**a**) Immunohistochemical analyses of NF-κB (magnification: 100× and scale bar: 50 μm). (**b**) Immunofluorescence analysis of NF-κB p65 in the jejunum (magnification: 400×; scale bars: 20 μm). (**c**–**e**) The mRNA expression levels of TNF-α, IL-1β, and IL-6 in the jejunum were compared (*n* = 6). (**f**,**g**) Western blot images representative of and quantification of p-NF-κB p65 and NF-κB p65 (*n* = 3). DAPI was used as the nuclear counterstain. Values are presented as the mean ± SD of each treatment. “^##^” *p* < 0.01 compared to control group; “*” *p* < 0.05 and “**” *p* < 0.01 compared ZEA group.

In order to better understand how RSV impacts the regulation of gut inflammation, the expression of various inflammatory cytokine mRNAs (such as IL-1b, IL-6, and TNF-α) was assessed using qRT-PCR. Jejunum inflammatory cytokine genes (IL-1b, IL-6, and TNF-α) were found to be increased after the ZEA challenge in comparison to the control mice, as illustrated in Figure 6c–e. However, RSV pretreatment following ZEA stimulation showed downregulated expression of these jejunum inflammatory cytokine genes.

Due to their significance in inflammation, the essential proteins of the NF-κB pathway were identified using Western blot analysis. Compared to the control group, the levels of p-NF-κB p65 and NF-κB p65 were notably elevated in the jejunum of mice exposed to ZEA, suggesting activation of the NF-κB pathway (Figure 6f,g).

We examined the subcellular location of the active subunit p65 to confirm the activation of NF-κB. RSV suppressed the ZEA-induced nuclear translocation of p-p65 in the jejunum, as indicated by the immunofluorescence results (Figure 6b). We also performed immunohistochemical analysis, which showed the same phenomenon described above (Figure 6a). Our findings collectively suggest that NF-κB plays a crucial role in how RSV works to decrease ZEA-induced intestinal inflammation.

### 3.7. RSV Reduces the Oxidative Stress Caused by ZEA through the Nrf2 Pathway

**Figure 7:**
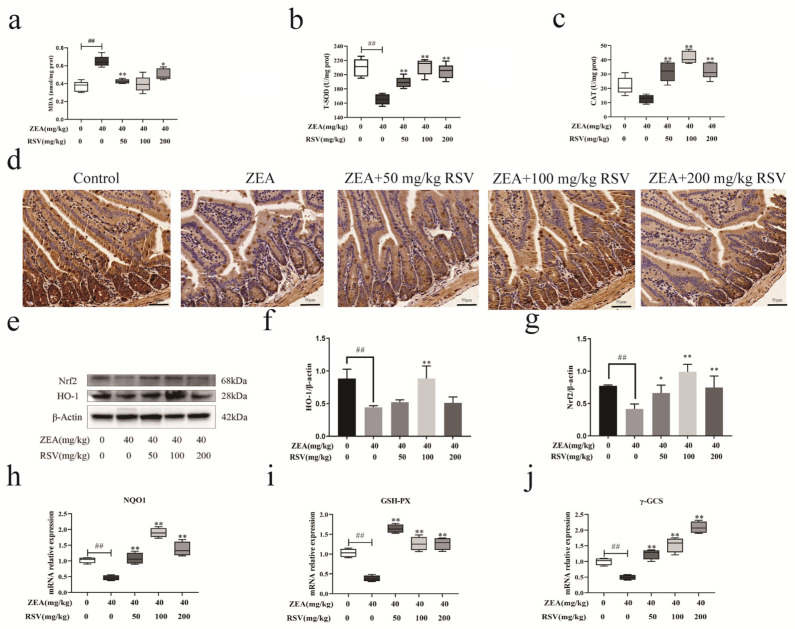
Impact of RSV on the Nrf2 signalling pathway in zearalenone-induced gut oxidative stress in mice. Indicators of oxidative stress (**a**) MDA, (**b**) T-SOD, and (**c**) CAT (*n* = 6). (**d**) Immunohistochemical analyses of Nrf2 (magnification: 100× and scale bar: 50 μm). Representative western blot images (**e**) and quantification of (**f**) HO-1 and (**g**) Nrf2 (*n* = 3). Relative expression of mRNA in the jejunum (**h**) NQO1, (**i**) GSH-PX, and (**j**) γ-GCS (*n* = 6). Values are presented as the mean ± SD of each treatment. “^##^” *p* < 0.01 compared to control group; “*” *p* < 0.05 and “**” *p* < 0.01 compared ZEA group.

ELISA was used to evaluate the MDA, T-SOD, and CAT levels in the intestinal tissue homogenate to study how RSV impacts jejunal oxidation-related enzyme activities. ZEA stimulation led to increased MDA production in the intestines compared to the control group, but supplementation with RSV notably lowered MDA levels (Figure 7a). Enzymatic activities in the intestine were evaluated by measuring T-SOD and CAT levels, which notably decreased after exposure to ZEA. However, their enzymatic activities were increased by the RSV pre-treatment.

Since the Nrf2 pathway regulates antioxidant enzyme expression, the potential mechanism of RSV on ZEA-induced jejunal oxidative damage was investigated by assessing Nrf2 protein expression and distribution. Thus, the impact of RSV on the movement of Nrf2 into the cell nucleus was observed using immunohistochemistry. The findings indicated that ZEA caused a slight movement of Nrf2 to the nucleus. At the same time, treatment with RSV notably enhanced the movement of Nrf2 to the nucleus in intestinal epithelial cells exposed to ZEA (Figure 7b). Furthermore, RSV significantly elevated the protein levels of Nrf2 and HO-1 in the mice jejunum, as demonstrated by Western blot analysis (Figure 7e–g). ZEA caused a notable reduction in the mRNA levels of NQO1, γ-GCS, and GSH-PX compared to the control group. In contrast, the addition of RSV prevented the ZEA-induced downregulated mRNA expression of NQO1, γ-GCS, and GSH-PX (Figure 7h–j). These findings indicate that RSV reduces the oxidative damage caused by ZEA in the gut through the Nrf2 pathway.

## 4. Discussion

ZEA is found in large quantities in mouldy feed materials and human food. Animals that consume feed contaminated with ZEA or humans who consume food contaminated with ZEA can develop serious health problems [24]. The upper part of the intestine absorbs these mycotoxins after ingestion. Since the epithelium of the intestine is the first line of defence against mycotoxins, this organ is likely to be exposed to higher concentrations of mycotoxins than other tissues [25]. The intestine is the primary target organ of mycotoxin toxicity, with its primary role of maintaining intestinal homeostasis. It is, therefore, essential to understand the mycotoxin-induced disruption of the intestinal barrier. RSV, a non-flavonoid polyphenolic compound, is found in several plants, including *Vitis vinifera*, *Arachis hypogaea*, and *Polygonum cuspidatum* [26]. Research has demonstrated its effectiveness as a powerful antioxidant and anti-inflammatory substance in laboratory and live animal studies [20,21]. RSV mitigates damage to the intestinal barrier in piglets treated with diquat, as shown by decreased production of inflammatory cytokines and enhanced antioxidant capacity in the jejunum [27]. RSV has been shown to attenuate ammonia exposure-induced oxidative stress and tissue damage in carp gills and livers by activating the Nrf2/HO-1 pathway and inhibiting the NF-κB pathway [28]. To date, the effect of RSV on ZEA-induced intestinal mucosal barrier disruption has not been the subject of investigation. RSV was discovered in this research to protect against ZEA-induced intestinal barrier impairment. We found that RSV treatment improves intestinal TJ protein abundance, alleviates intestinal inflammation, and reduces oxidative damage under ZEA stimulation conditions, enhancing intestinal barrier function. In addition, we were able to elucidate the underlying mechanism by which RSV protects the intestinal barrier. The present study improves our understanding of the effect of RSV on the regulation of the homeostasis of the intestinal barrier.

Increased intestinal permeability is an indicator of intestinal barrier dysfunction. It is widely recognised that increased intestinal permeability is an underlying pathogenic factor. Numerous factors, such as the gut microbiome, mucosal layer, integrity of epithelial cells, junctions between epithelial cells, immune responses, vascular system of the intestines, and movement of the intestines, can affect gut permeability [29]. DAO and D-lactate have traditionally been the two significant biomarkers used to evaluate gut cell damage and function. When the intestinal barrier function is impaired, these two biomarkers are released into the bloodstream [30]. This experiment produced similar results, confirming that ZEA toxins accelerate DAO release into the bloodstream and damage gut structure and function. Following pretreatment with RSV, serum levels of DAO were notably reduced compared to the ZEA group. RSV can reduce the permeability of intestinal mucosa and effectively alleviate the impairment of intestinal barrier function caused by ZEA.

A prerequisite for maintaining intestinal barrier function is the integrity of the intestinal structure. Alterations in the structure of the intestines are viewed as vital signs of how the intestines react to harmful substances, such as the length of the villi, the depth of the crypts, and the ratio of villus length to crypt depth [31]. RSV in this research notably improved ZEA-induced villus shortening and thinning, decreased the ratio of villus length to crypt depth, and disturbed TJs among intestinal epithelial cells, as shown by changes in the morphology of intestinal villi seen through H&E staining and TEM. However, RSV may reduce ZEA-induced damage through the improvement of intestinal barrier integrity and morphology.

A thick layer of mucus covers the intestinal epithelium, aiding intestinal homeostasis regulation [32]. Goblet cells are responsible for the production of intestinal mucus. It is a highly organised network of glycoproteins consisting mainly of MUC-2 [33]. Intestinal infections caused by bacteria, viruses, and harmful chemicals can affect goblet cell responses and MUC-2 production, disrupting the intestinal mucosal barrier [34]. ZEA induced a notable decrease in the number of goblet cells and the levels of MUC-2 mRNA in the jejunum of mice in the research. However, RSV supplementation reversed the reduction in goblet cell number and MUC-2 mRNA levels in the gut.

TJs are crucial for preserving the intestinal barrier’s integrity and controlling epithelial cell permeability. TJ proteins are mainly claudins, occludins, and the ZO family [35,36]. Dysregulation of TJ proteins can cause a rise in the specific permeability of the intestinal epithelium, leading to a breakdown in the physical barrier and contributing to the development of intestinal disorders. It was found that combined exposure of Caco-2 cells to acrylamide and ochratoxin A produced synergistic toxicity and disrupted the intestinal barrier by reducing TJ protein expression [37]. The current research found reduced occludin, claudin-1, and ZO-1 expression levels in the intestines of mice exposed to ZEA. Nevertheless, RSV inhibited the decreases in occludin, claudin-1, and ZO-1 caused by ZEA. These findings indicate that RSV could protect intestinal integrity following the ZEA challenge by regulating intracellular TJs.

Oxidative stress is a potential mechanism of intestinal barrier dysfunction and has been implicated in the pathogenesis of gastrointestinal diseases [38]. Oxidative stress, partially regulated by inhibiting antioxidant enzyme activity and increased lipid peroxidation, is an underlying mechanism of ZEA-induced intestinal inflammation and intestinal barrier dysfunction [39]. The current study’s results align with these findings, indicating that ZEA decreased CAT and T-SOD levels while raising MDA accumulation in the intestines. In the meantime, supplementation of RSV enhanced antioxidant functions and decreased intestinal MDA buildup in mice exposed to ZEA. The Nrf2 signalling pathway is crucial in regulating tissue damage caused by oxidative stress. Activating the Nrf2 pathway may shield the intestinal tissue from oxidative harm [40,41]. Nrf2 is a crucial modulator of HO-1 expression. Under oxidative stress conditions, Nrf2 moves from the cytoplasm to the nucleus, triggering the expression of various genes responsible for antioxidant enzymes like NQO1, HO-1, and GSH. In the present study, RSV increased the levels of Nrf2 and its nuclear localisation in the jejunum of mice exposed to ZEA. Furthermore, this was accompanied by a ZEA-induced decrease in NQO1, GSH-PX, and γ-GCS gene expression levels, all reversed by RSV supplementation. The above results suggest that RSV induces the activation of the Nrf2 signalling pathway, which reduces the oxidative damage caused by ZEA in the jejunum. These findings indicate that RSV promotes the activation of Nrf2 signalling to attenuate oxidative damage induced by ZEA in the jejunum.

Inflammation is an essential regulator of intestinal mucosal immune barrier function [42]. NF-κB is crucial in controlling the gut barrier as a traditional pathway for immunity and inflammation [43]. The NF-κB pathway regulates inflammation cytokines. NF-κB is mainly found in the cytoplasm under normal conditions. Their binding to IκB results in the inhibition of its transcriptional activity, forming an inactive complex. The receptors on cells are triggered when the body is exposed to external substances or pro-inflammatory cytokines. Phosphorylation and the degradation of IκB result in NF-κB activation. NF-κB triggers the production of inflammatory cytokines [44]. Growing evidence indicates that overproduction of inflammatory cytokines (IL-1β, IL-6, and TNF-α) leads to heightened local inflammation and results in tissue harm, such as injury to the intestinal barrier. Moreover, RSV suppressed NF-κB activation and the resulting gene expression triggered by a ZEA in Caco-2 cells [20]. RSV pretreatment inhibited the ZEA-induced rise in NF-κB phosphorylation and notably reduced IL-1β, IL-6, and TNF-α levels, consistent with earlier research. Thus, RSV mitigates ZEA-induced damage to the intestinal barrier by regulating the inflammatory response in the intestines through inhibition of the NF-κB pathway.

## 5. Conclusions

In conclusion, supplementation with RSV at different concentrations could effectively ameliorate ZEA-induced intestinal damage in mice. The study findings indicated that the most effective protection was achieved by including 100 mg of RSV. Further research showed that RSV’s protection against ZEA-induced intestinal dysfunction is linked to Nrf2 pathway activation and NF-κB pathway inhibition. These results not only provide a new theoretical basis for the prevention of ZEA-induced intestinal damage but also provide prospects for the application of RSV in food, agricultural, and pharmaceutical fields.

## Figures and Tables

**Table 1:** Sequences of Primer Used for qRT-PCR.

Gene Name	Primer Sequences (5′ to 3′)	Product Length (bp)
GAPDH	TGAACGGGAAGCTCACTGG	307
	TCCACCACCCTGTTGCTGTA	
MUC-2	AGGGCTCGGAACTCCAGAAA	106
	CCAGGGAATCGGTAGACATCG	
NQO1	CATTGCAGTGGTTTGGGGTG	111
	TCTGGAAAGGACCGTTGTCG	
γ-GCS	TGGATGATGCCAACGAGTC	185
	CCTAGTGAGCAGTACCACGAATA	
GSH-PX	GAAGTGCGAAGTGAATGG	224
	TGTCGATGGTACGAAAGC	
Occludin	GCAATGACATGTATGGCGGA	162
	GGCGACGTCCATTTGTAGAAG	
Claudin-1	TGGGGCTGATCGCAATCTTT	158
	CCCAATGACAGCCATCCACA	
ZO-1	GCATTATTCGCCTTCATACA	230
	GCTCCATACCAACCATCAT	
IL-1β	TGCCACCTTTTGACAGTGATG	118
	TGTGCTGCTGCGAGATTTGA	
IL-6	TGATGGATGCTACCAAACTGG	97
	GTGACTCCAGCTTATCTCTTGG	
TNF-α	TTCTCATTCCTGCTTGTGGCA	179
	CTCCACTTGGTGGTTTGTGAG	

## Data Availability

The original contributions presented in the study are included in the article, further inquiries can be directed to the corresponding author.

## Abbreviations

BSA, bovine serum albumin; BW, body weight; CAT, catalase; DAB, diaminobenzidine; DAO, diamine oxidase; DAPI, 4′,6-diamidino-2-phenylindole; GSH-PX, glutathione peroxidase; H&E, hematoxylin and eosin; HO-1, heme oxygenase-1; IL-1β, interleukin-1β; IL-6, interleukin-6; IκB, inhibitor kappa B alpha; MDA, malondialdehyde; MUC-2, mucin-2; NF-Κb,p65, nuclear factor-kappaB; NQO1, nad(p)h quinone oxidoreductase-1; Nrf2, nuclear factor E2-related factor 2; PAS, periodic acid-schiff; PBS, phosphate buffer solution; p-NF-κB,pp-65; phospho-nuclear factor-kappaB; ROS, reactive oxygen species; RSV, resveratrol; TEM, transmission electron microscopy; TJ, tight junction; TNF-α, tumor necrosis factor-α; T-SOD, total superoxide dismutase; ZEA, zearalenone; ZO-1, zonula occludens-1; γ-GCS, γ-glutamylcysteine synthetase.
