# Correction: Xia et al. Resveratrol Alleviates Zearalenone-Induced Intestinal Dysfunction in Mice through the NF-κB/Nrf2/HO-1 Signalling Pathway. *Foods* 2024, *13*, 1217

**DOI:** 10.3390/foods13111686

**Published:** 2024-05-28

**Authors:** Sugan Xia, Chaoyue Yan, Jianhong Gu, Yan Yuan, Hui Zou, Zongping Liu, Jianchun Bian

**Affiliations:** 1 College of Veterinary Medicine, Yangzhou University, Yangzhou 225009, Chinaliuzongping@yzu.edu.cn (Z.L.); 2 Jiangsu Co-Innovation Center for Prevention and Control of Important Animal Infectious Diseases and Zoonoses, Yangzhou 225009, China; 3 Joint International Research Laboratory of Agriculture and Agri-Product Safety, Ministry of Education of China, Yangzhou University, Yangzhou 225009, China

In the original publication [1], there was a mistake in “Figure 7. Impact of RSV on the Nrf2 signalling pathway in zearalenone-induced gut oxidative stress in mice”. The research images were duplicated due to carelessness in drawing the combined images, resulting in ZEA + 50 mg/kg RSV and ZEA + 200 mg/kg RSV being the same (Figure 7d). We have corrected Figure 7d on the basis that the scientific conclusions are unaffected. The corrected Figure 7 is as follows:

This correction has been approved by the Academic Editor. The original publication has also been updated.

## Figures and Tables

**Figure 7:**
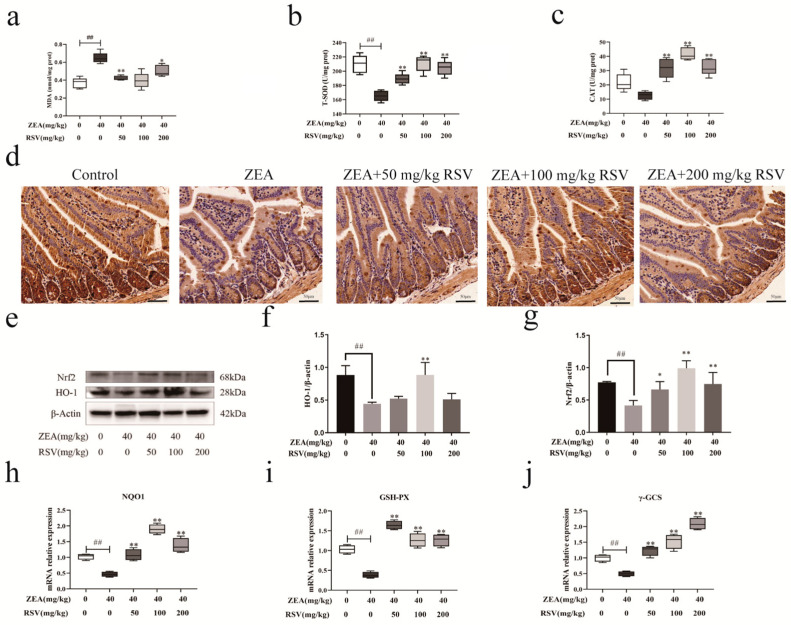
Impact of RSV on the Nrf2 signalling pathway in zearalenone-induced gut oxidative stress in mice. Indicators of oxidative stress (**a**) MDA, (**b**) T-SOD, and (**c**) CAT (*n* = 6). (**d**) Immunohistochemical analyses of Nrf2 (magnification: 100× and scale bar: 50 μm). Representative western blot images (**e**) and quantification of (**f**) HO-1 and (**g**) Nrf2 (*n* = 3). Relative expression of mRNA in the jejunum (**h**) NQO1, (**i**) GSH-PX, and (**j**) γ-GCS (*n* = 6). Values are presented as the mean ± SD of each treatment. “^##^” *p* < 0.01 compared to control group; “*” *p* < 0.05 and “**” *p* < 0.01 compared ZEA group.
